# Enhanced Antibody Production in Clever-1/Stabilin-1–Deficient Mice

**DOI:** 10.3389/fimmu.2018.02257

**Published:** 2018-10-08

**Authors:** Johannes Dunkel, Miro Viitala, Marika Karikoski, Pia Rantakari, Reetta Virtakoivu, Kati Elima, Maija Hollmén, Sirpa Jalkanen, Marko Salmi

**Affiliations:** ^1^MediCity Research Laboratory, University of Turku, Turku, Finland; ^2^Institute of Biomedicine, University of Turku, Turku, Finland

**Keywords:** humoral immune responses, scavenging receptors, macrophages, B cells, immunoglobulins

## Abstract

Clever-1, encoded by the *Stab1* gene, is a scavenger and leukocyte trafficking receptor expressed by subsets of vascular and lymphatic endothelial cells and immunosuppressive macrophages. Monocyte Clever-1 also modulates T cell activation. However, nothing is known about the possible links between B cell function and Clever-1. Here, we found that *Stab1* knockout mice (*Stab1*^−/−^) lacking the Clever-1 protein from all cells present with abnormally high antibody levels under resting conditions and show enhanced humoral immune responses after immunization with protein and carbohydrate antigens. Removal of the spleen does not abolish the augmented basal and post-immunization antibody levels in Clever-1–deficient mice. The increased IgG production is also present in mice in which Clever-1 is selectively ablated from macrophages. When compared to wildtype macrophages, Clever-1–deficient macrophages show increased TNF-α synthesis. In co-culture experiments, monocytes/macrophages deficient of Clever-1 support higher IgM production by B cells, which is blocked by TNF-α depletion. Collectively, our data show that the excessive inflammatory activity of monocytes/macrophages in the absence of Clever-1 results in augmented humoral immune responses *in vivo*.

## Introduction

Clever-1 (common lymphatic endothelial and vascular endothelial receptor-1), a large 280 kDa multidomain glycoprotein receptor encoded by the *Stab1* gene, is associated with scavenging and leukocyte trafficking (1). Clever-1 (also known as stabilin-1 and Feel-1) is expressed by lymphatic vessels and non-continuous vascular endothelial cells in various organs such as the spleen, bone marrow, and liver, as well as by subsets of immunosuppressive macrophages (2–5). Clever-1 is a scavenger receptor with multiple ligands, including SPARC (secretory protein, acidic, and rich in cysteine), placental lactogen and oxidized, and acetylated low-density lipoprotein (6–10). The ability of Clever-1 to modulate angiogenesis and to bind bacteria in transfected cell lines has also been reported (4). Moreover, Clever-1 on lymphatic and vascular endothelia is involved in leukocyte—endothelium interactions (5, 11–14). In macrophages, Clever-1 supports their adhesion to the vascular endothelium under shear stress (15). In humans, Clever-1^high^ monocytes display an anti-inflammatory gene signature and induce lower γ interferon (IFNγ) and tumor necrosis factor-α (TNF-α) production in T cell antigen recall assays when compared to Clever-1^low^ monocytes (16).

In contrast to macrophage and T cell biology, the potential functions of Clever-1 during humoral immune responses remain completely unknown. In addition to reactive antibodies against newly encountered antigens, humoral immunity also includes natural antibodies and antibodies from previous infections or vaccinations (17, 18). Natural T cell–independent (TI) antibodies are mainly produced by marginal zone B cells (MZB) in the spleen and B1 cells in the pleural and peritoneal cavities (19–21). Classical T cell—dependent (TD) antibody responses against protein antigens, in contrast, require concurrent activation of CD4^+^ helper T cells and B cells in lymphoid organs (22).

The aim of this work was to study the potential effects of Clever-1 on humoral immune responses. For that purpose, we evaluated natural and elicited TI and TD antibody responses, antigen scavenging and the collaboration of B cells with wildtype and Clever-1–deficient monocytes/macrophages *in vivo* and *in vitro*. We observed that the enhanced inflammatory activity of Clever-1–deficient monocytes/macrophages is associated with augmented natural and induced antibody production.

## Materials and methods

### Mice

All animal work was conducted in the Central Animal Laboratory, University of Turku, Turku, Finland. Clever-1 knockout (*Stab1*^−/−^) and macrophage-specific *Lyz2*-*Cre*/*Stab1*^fl/fl^ mice have been previously described (16). Age-matched wildtype, *Stab1*^−/−^ AND *Lyz2*-*Cre*/*Stab1*^fl/fl^ mice were used in all experiments. All experiments were performed at least in duplicate, with a minimum of three mice per group, and the displayed results were pooled whenever possible. Mice were typically 6–8 weeks old at the beginning of experiments.

### Immunizations

NP-Ficoll (conjugation ratio 4:9) and NP-KLH (conjugation ratio 2:3, both from Biosearch Technologies) were dissolved in sterile PBS (Sigma). 25 μg of NP-Ficoll in 200 μl PBS or 100 μg of NP-KLH in 200 μl of PBS/alum adjuvant (mixed at a 1:1 ratio, from Thermo Fisher) was injected into mice intraperitoneally. Blood samples were collected into heparin-coated capillaries by tail vein punctures on indicated days. Plasma was separated by centrifugation and frozen. In certain experiments, the mice were sacrificed 7 days after immunization and tissues were collected for analyses.

### Analysis of antibody responses

Total IgM and IgG levels were measured with commercial ELISA kits from Thermo Fisher, according to manufacturer's instructions.

(4-hydroxy-3-nitrophenyl)acetyl (NP)-specific IgG, IgM and IgG3 levels were measured with indirect ELISA, which was optimized in-house. Briefly, Nunc MaxiSorp plates were coated overnight with NP-BSA (conjugation ratio 4:1, from Biosearch Technologies) and blocked with 1% BSA in PBS at RT for 2 h. Samples were diluted in 1% BSA in PBS, added to the plates in duplicate and incubated at +4°C overnight. Pooled plasma from immunized mice was used as standards for concentration curves. Polyclonal HRP-conjugated secondary antibodies (anti-mouse IgM, IgG, and IgG3, all from Southern Biotech) were diluted in 1% BSA in PBS, added to the plates and incubated at RT for 2 h. NP-reactive antibodies were visualized by adding TMB chromogen (Invitrogen) and the reaction was stopped with 0.2 M hydrochloric acid. Absorbance was measured with Tecan Infinite M200 and analyzed with the Magellan software (both from Tecan).

### Antibodies and flow cytometry

Leukocytes from the spleen and bone marrow were isolated using mechanical teasing as described (23). Cells from the peritoneal cavity were obtained by peritoneal lavage. Blood leukocytes were analyzed from the EDTA-anticoagulated whole blood.

The following fluorochrome-conjugated monoclonal anti-mouse antibodies from BD were used: α-CD4-APC-Cy7, α-CD5-FITC, α-CD8α-PerCP-Cy5.5, α-CD11b-PE, α-CD19-FITC, α-CD21/35-PE, α-CD23-BV510, α-CD24-BV650, α-CD43-BV605, α-CD44-PerCP-Cy5.5, α-CD45-PE, α-B220-BV421, α-CD62L-Alexa Fluor 647, α-CD138-BV510, α-Fas-PE, α-fluorescein-Alexa Fluor 488, α-IgM-APC-Cy7, α-IgD-Alexa Fluor 647, and α-Ly6C-BV421. Regulatory T cells were stained using the Mouse Regulatory T Cell Staining Kit 1 (eBioscience). The mStab1–1.26 (24) and isotype-matched control MOPC-21 (Bio X Cell) antibodies were conjugated with the Alexa Fluor 647 Antibody Labeling Kit according to the manufacturer's instructions (Thermo Fisher). Fc receptors were blocked with mouse Fc Block (BD) before staining with specific antibodies. Samples were run on LSRII or LSRFortessa (both from BD) and analyzed with the FlowJo software version 10 (Treestar).

The specific lymphocyte populations were gated as follows: MZB: CD19^+^ CD21/CD35^high^ CD23^low^. FoB: CD19^+^ CD21/CD35^low^ CD23^high^. GCB: B220^+^ Fas^high^. Plasma cells: CD45^+^ B220^low^ CD138^high^. B1 cells: CD19^+^ CD5^+/−^ CD11b^+^. Pre-pro B cells: B220^+^ CD24^low^ CD43^+^. Pre-B cells: B220^+^ CD24^+^ CD43^−^ IgM^−^ IgD^−^. Immature B cells: B220^+^ CD24^+^ CD43^−^ IgM^low^ IgD^−^. Transitional B cells: B220^+^ CD24^+^ CD43^−^ IgM^+^ IgD^−^. Early mature B cells: B220^+^ CD24^+^ CD43^−^ IgM^+^ IgD^+^. Late mature B cells: B220^+^ CD24^+^ CD43^−^ IgM^low^ IgD^+^. Naïve T cells: CD4^+^/CD8^+^ CD44^low^ CD62L^high^. Central memory T cells: CD4^+^/CD8^+^ CD44^high^ CD62L^high^. Effector T cells: CD4^+^/CD8^+^ CD44^high^ CD62L^low^. Regulatory T cells: CD4^+^ CD25^+^ FoxP3^+^. Isotype-matched negative control antibodies conjugated to the same fluorochrome were used to determine gating.

### Anti-clever-1 uptake

Blood was collected by cardiac puncture and the red blood cells removed with Pharm Lyse lysing solution (BD). Leukocytes were incubated at 37°C for 1 h with 20 μg/ml of either mStab1–1.26- or MOPC-21-Alexa Fluor 647 (conjugated as described above), washed, stained with α-CD11b-PE and α-Ly6C-BV421 and analyzed by flow cytometry. The geometric mean fluorescence intensities of the Ly6C^high^ and Ly6C^low^ monocyte populations of wildtype mice were normalized to the unspecific antibody binding of the corresponding populations of *Stab1*^−/−^ mice, which were set at 100%.

### Microscopy

Eight micrometer cryosections were cut and fixed in −20°C acetone for 5 min. Primary unconjugated or biotin-conjugated antibodies against CD169, CD209b, F4/80, Meca32 (anti-PV-1) and 9–11 (anti-Clever-1) (11) were diluted in 2% plasma in PBS. Species-specific polyclonal secondary antibodies or streptavidin conjugated with Alexa Fluor 488 or Alexa Fluor 546 (Life Technologies) diluted 1:1,000 in PBS were used for detection as appropriate. In certain experiments, Alexa Fluor 647–conjugated Meca32 was used. Imaging was performed with an Olympus BX60 wide-field microscope or a Zeiss LSM780 confocal microscope equipped with a motorized stage. Images were analyzed with the ImageJ software version 1.50b (NIH) or the Imaris software version 8 (Bitplane). Fluorescence values were thresholded and the background was subtracted with reference to isotype control staining.

Hematoxylin/eosin–stained spleen paraffin sections were scanned with a Panoramic 250 Slide Scanner and analyzed with the CaseViewer software (both from 3DHISTEC).

Cells were cultured *in vitro* on coverslips and fixed with 4% paraformaldehyde and permeabilized and blocked with 0.1% Triton X in PBS with 30% horse serum for 20 min. After three washes with PBS, α-TNF-α-Alexa Fluor 488 (Thermo Fisher), and mStab1.26-Alexa Fluor 647 (conjugated as described above) antibodies diluted 1:200 were added for 1 h. After washes, nuclei were stained with Hoechst. Coverslips were mounted with Vectashield mounting medium. Confocal 3D images were captured with Zeiss Axiovert 200 M with the spinning disk confocal unit Yokogawa CSU22 and the Zeiss Plan-Neofluar 63 × oil/1.4 NA objective. Images were analyzed with the ImageJ software.

### Splenectomy

Mice were anesthetized with intraperitoneal ketamine (Orion Pharma) and xylazine (Bayer). Buprenorphine (Orion Pharma) was injected subcutaneously for perioperative analgesia. The peritoneal cavity was opened at the left flank and the spleen was removed by thermo-cauterization. The peritoneum and skin were closed with absorbable suture material. Carprophen was injected subcutaneously once daily for postoperative analgesia.

### Leukocyte enumeration

Blood was drawn by tail vein puncture 30 days after splenectomy or sham surgery and peripheral blood cell populations were quantified with a VetScan hematology system (Abaxis).

### *In vivo* antigen capture

NP-AECM-Ficoll-fluorescein (conjugation ratio 94:11, from Biosearch Technologies) was diluted in PBS and injected intravenously. Mice were sacrificed 45 min or 7 days after the injections and the spleens were collected and frozen in OCT (Sakura) for sectioning.

### M2 macrophage culture

Bone marrow cells from adult wildtype and *Stab1*^−/−^ mice were cultured in IMDM supplemented with 10% FCS, 1% penicillin/streptomycin and 20 ng/ml M-CSF (PeproTech). On day 4, dexamethasone was added to 100 nM and the cells cultured for an additional 3 days. To induce TNF-α production, LPS was added to 50 ng/ml for 5 h. The cells were treated with 10 μg/ml of brefeldin A to inhibit cytokine secretion.

### Co-culture experiments

Splenocytes and bone marrow cells were collected as described above and passed through Lympholyte-M density gradients to remove dead cells (Cedarlane). Splenic B cells were isolated with the B Cell Isolation Kit (mouse), from which CD138^+^ plasma cells were depleted with CD138 Microbeads (mouse) according to the manufacturer's instructions. Monocytes were isolated with the Monocyte Isolation Kit (Bone Marrow) (mouse) according to the manufacturer's instructions (all kits were from Miltenyi Biotec). Purity of the isolated cells was assessed by flow cytometry. Isolated B cells were 90% CD45R^+^ CD138^−^ and isolated monocytes 90% CD11b^+^ Ly6C^high^. Isolated cells were plated at 0.5 × 10^6^ cells/ml in RPMI-1640 supplemented with 10% FCS, 1% penicillin/streptomycin and 1% Glutamax in the indicated combinations and incubated for 5 days, after which the supernatants were collected for ELISA (described above). The neutralizing α-TNF-α antibody clone XT3.11 (Bio X Cell) was used at 50 μg/ml.

### RNA sequencing and quantitative PCR

Total RNA from dissected livers of E17.5 and 2-weeks-old wildtype (*n* = 4 for both time points) and *Stab1*^−/−^ (*n* = 3 for E17.5 and 4 for 2 weeks) mice was isolated and RNA-seq performed as described in (25). The RNA-seq results have been deposited into the Gene Expression Omnibus database (accession number GSE114969). For qPCR, total RNA was isolated from adult wildtype and *Stab1*^−/−^ mouse spleens (*n* = 3) using the RNeasy Kit (Qiagen) and reverse transcribed to cDNA with the SuperScript VILO cDNA Synthesis Kit (ThermoFisher Scientific) according to the manufacturers' instructions. Quantitative PCR was carried out using Taqman Gene Expression Assays (Thermo Fisher) for *Ifi202b* (Mm00839397_m1; target gene), and *Actb* (Mm00607939_s1; control gene). The reactions were run using a QuantStudio12K Flex Real-Time PCR system (Thermo Fisher) at the Finnish Microarray and Sequencing Centre, Turku Centre for Biotechnology. Relative quantification was performed using the 2^−ΔΔ*CT*^ method, in which the gene expression level in knockout mice is compared to that in wildtype mice, in which RQ = 1.

### Statistical analyses

Experimental data are presented as scatter plots with bars representing the means ± standard errors of the means (s.e.m.), excepting kinetic experiments for NP-specific antibody titers, which are presented as means ± s.e.m. Probabilities were calculated with Student's unpaired two-tailed *t*-test with Welch's correction when applicable or with two-way ANOVA followed by Bonferroni's multiple comparisons tests using GraphPad Prism 7 (GraphPad Software). Threshold of statistical significance was set at *P* < 0.05.

## Results

### Elevated plasma IgM and IgG levels in the absence of Clever-1

**Figure 1:**
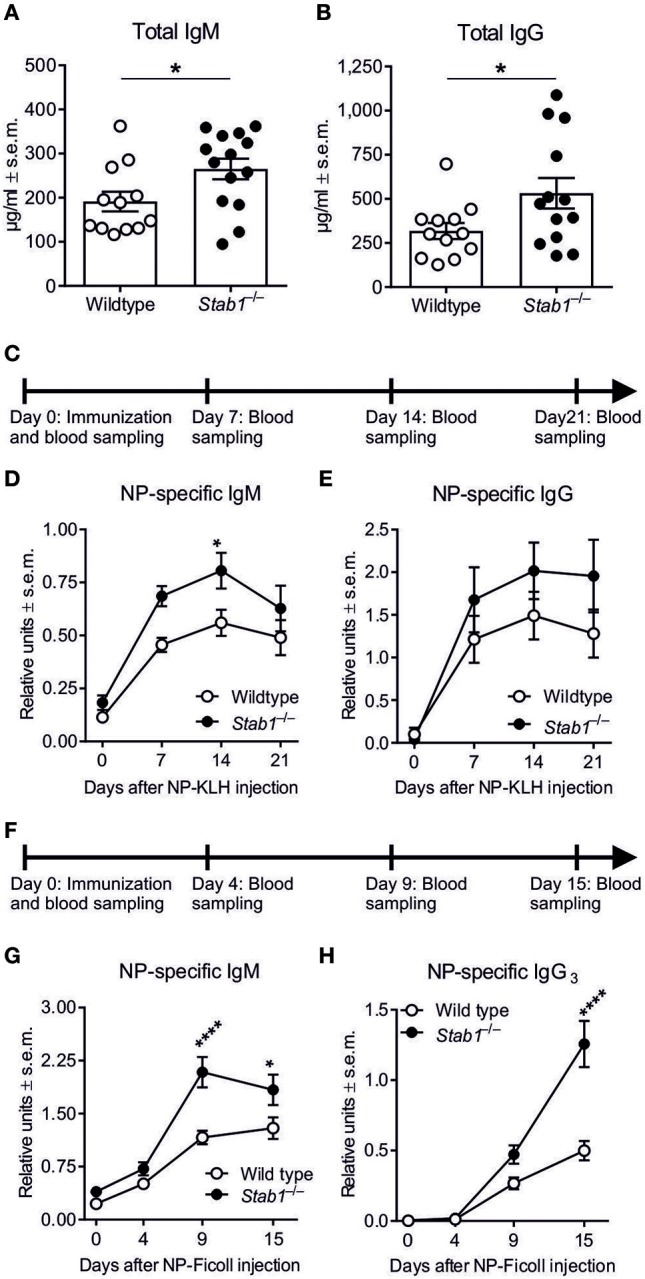
*Stab1*^−/−^ mice have elevated antibody levels at baseline and after immunization with TD and TI antigens. **(A,B)** Baseline plasma concentrations of IgM **(A)** and IgG **(B)** as determined by ELISA. **(C)** Experimental protocol for NP-KLH (TD antigen) immunization. **(D,E)** Kinetics of NP-specific IgM **(D)**, and IgG **(E)** production after NP-KLH immunization (RU, relative units) determined by ELISA. **(F)** Experimental protocol for NP-Ficoll (TI-2 antigen) immunization. **(G–H)** Kinetics of NP-specific IgM **(G)** and IgG_3_ **(H)** production after NP-Ficoll immunization in wildtype and *Stab1*^−/−^ mice. **(A,B)** Each data point represents one mouse and bars represent the means ± s.e.m., *n* = 12 (wildtype) and 14 (*Stab1*^−/−^) **(A)** and 12 (wildtype) and 13 (*Stab1*^−/−^) **(B)**. Statistical significance was determined with Student's unpaired two-tailed *t*-test **(A)** and Student's unpaired two-tailed *t*-test with Welch's correction **(B)**, ^*^P < 0.05. **(D,E,G,H)** Data are presented as means ± s.e.m., *n* = 10–11 (wildtype) and 8–9 (*Stab1*^−/−^) **(D, E)**, 12 (wildtype) and 15 (*Stab1*^−/−^) **(G)** and 4 (wildtype) and 5 (*Stab1*^−/−^) **(H)**. Statistical significance was determined with two-way ANOVA followed by Bonferroni's multiple comparisons tests, ^*^P < 0.05, ^****^P < 0.0001.

The role of Clever-1 in B cell biology has not been previously studied. Therefore, we initially measured the baseline plasma concentrations of IgM and IgG in wildtype and *Stab1*^−/−^ mice that lack Clever-1 from all cells. ELISA revealed that the baseline levels of total IgM were increased by 38.7 ± 5.4% and total IgG by 67.2 ± 14.0% in *Stab1*^−/−^ mice compared to wildtype mice (*P* = 0.0327 and 0.0425, respectively) (Figures 1A,**B**). Thus, the genetic deletion of Clever-1 resulted in constitutively increased basal levels of both IgM and IgG *in vivo*.

### Accelerated TD and TI-2 antibody responses in *Stab1*^−/−^ mice

To study the role of Clever-1 in classical TD antibody production, we used intraperitoneal nitrophenyl (NP)-KLH immunization with adjuvant (Figure 1C). Kinetic measurements of plasma antibody levels showed that the NP-KLH–specific IgM response was accelerated in *Stab1*^−/−^ mice compared to wildtype mice (Figure 1D). On day 7, the level of NP-specific IgM was 0.46 ± 0.034 relative units (RU) in wildtype mice and 0.69 ± 0.048 RU in *Stab1*^−/−^ mice (*P* = 0.0742), and on day 14, the NP-specific IgM levels reached 0.56 ± 0.062 RU and 0.81 ± 0.084 RU in wildtype and *Stab1*^−/−^ mice, respectively (*P* = 0.0496) (Figure 1D). The level of NP-specific IgG was 52.8 ± 16.3% higher on day 21 after NP-KLH immunization in *Stab1*^−/−^ mice compared to wildtype mice, although the differences were not statistically significant at any measured time point (Figure 1E).

TI antibody responses were studied with the TI-2 antigen NP-Ficoll (Figure 1F). We observed that the NP-specific IgM response was more robust in *Stab1*^−/−^ mice compared to wildtype mice (Figure 1G). On day 9, the level of NP-specific IgM was 1.16 ± 0.10 RU in wildtype mice and 2.09 ± 0.22 RU in *Stab1*^−/−^ mice (*P* < 0.0001), and on day 15, the NP-specific IgM levels were 1.29 ± 0.15 RU in wildtype mice and 1.84 ± 0.22 RU in *Stab1*^−/−^ mice (*P* = 0.0335) (Figure 1G). Similarly, class switching to the NP-specific IgG_3_ isotype, which is relatively specific to TI-2 antibody responses (26), was enhanced in *Stab1*^−/−^ mice (Figure 1H). On day 15, the levels of NP-specific IgG_3_ remained at 0.50 ± 0.070 RU in wildtype mice but rose to 1.26 ± 0.16 RU in *Stab1*^−/−^ mice (*P* < 0.0001) (Figure 1H). Collectively, these data show that both TD and especially TI-2 antibody responses are enhanced in mice lacking Clever-1.

### Altered splenic B cell subpopulations in *Stab1*^−/−^ mice

**Figure 2:**
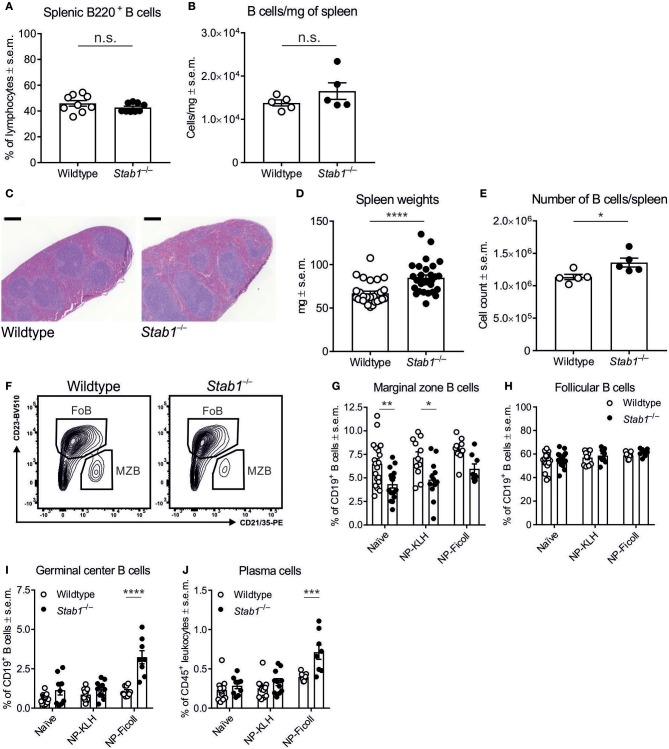
*Stab1*^−/−^ mice have enlarged spleens and altered B cell populations. **(A,B)** Frequencies of B220^+^ B cells in the spleen **(A)** and B cell number per mg of spleen **(B)** in wildtype and *Stab1*^−/−^ mice. **(C–E)** Representative hematoxylin/eosin–stained spleen sections (scale bar, 200 μm) **(C)**, spleen weights **(D)**, and the absolute numbers of B cells per spleen **(E)** in wildtype and *Stab1*^−/−^ mice. **(F–H)** Representative gating **(F)** and the frequencies of splenic MZB (CD19^+^ CD23^low^ CD21/CD35^+^) **(G)** and FoB (CD19^+^ CD23^+^ CD21/35^low^) **(H)** in naïve and NP-KLH- or NP-Ficoll-immunized mice. **(I,J)** Frequencies of splenic GCB (CD19^+^ Fas^+^) **(I)** and PC (CD45^+^ CD19^−^ CD138^high^) **(J)** naïve and NP-KLH- or NP-Ficoll-immunized mice. **(A,B,D,E,G–J)** Each data point represents one mouse and bars represent the means ± s.e.m., *n* = 9 (wildtype and *Stab1*^−/−^) **(A)**, 5 (wildtype and *Stab1*^−/−^) **(B)**, 28 (wildtype) and 29 (*Stab1*^−/−^) **(D)**, 5 (wildtype and *Stab1*^−/−^) **(E)**, 9–19 (wildtype) and 8–16 (*Stab1*^−/−^) **(G, H)** and 9–14 (wildtype) and 8–11 (*Stab1*^−/−^) **(I, J)**. Statistical significance was determined with Student's unpaired two-tailed *t*-test **(A,B,D,E)** or two-way ANOVA followed by Bonferroni's multiple comparisons tests **(G–J)**, ^*^P < 0.05, ^**^P < 0.01, ^***^P < 0.001, ^****^P < 0.0001, n.s. not significant.

Since the spleen plays a central role in humoral immunity, we next compared splenic B cell populations between wildtype and *Stab1*^−/−^ mice. The frequencies of B220^+^ B cells did not differ significantly between wildtype and *Stab1*^−/−^ mice, nor did their absolute numbers per mg of spleen (Figures 2A,B). Nevertheless, the spleens of *Stab1*^−/−^ mice were on average larger than those of wildtype mice (*P* < 0.0001), increasing the absolute number of B cells per spleen (Figures 2C–E). Unexpectedly, in spite of the increased natural TI antibody levels in *Stab1*^−/−^ mice, the frequency of MZB was reduced by 33.3 ± 4.0% in naïve *Stab1*^−/−^ mice compared to naïve wildtype mice (*P* = 0.0041) (Figures 2F,G). The frequencies of splenic follicular B cells (FoB) and CD4^+^ and CD8^+^ T cells were comparable between naïve wildtype and *Stab1*^−/−^ mice, although the frequency of naïve CD4^+^ T cells in the spleen was increased in *Stab1*^−/−^ mice compared to wildtype mice (*P* < 0.0001) (Figure 2H and Supplementary Figures S1A–C).

After immunization with NP-KLH, MZB remained scarcer by 32.5 ± 4.7% in *Stab1*^−/−^ mice compared to wildtype mice (*P* = 0.0147), but we did not observe statistically significant differences in the frequencies of FoB in response to either TD or TI antigen (Figure 2G and H). In contrast, after immunization with NP-Ficoll, the frequencies of reactive germinal center B cells (GCB) and plasma cells (PC) were robustly elevated by 203.4 ± 31.1% and 79.0 ± 9.9%, respectively, in *Stab1*^−/−^ mice compared to wildtype mice (*P* < 0.0001 and *P* = 0.0001, respectively) (Figures 2I,J). Thus, *Stab1*^−/−^ mice manifest with constitutive hyperimmunoglobulinemia in the absence of increased frequencies of MZB or FoB in the spleen.

### Extrasplenic factors contribute to hyperimmunoglobulinemia in *Stab1*^−/−^ mice

**Figure 3:**
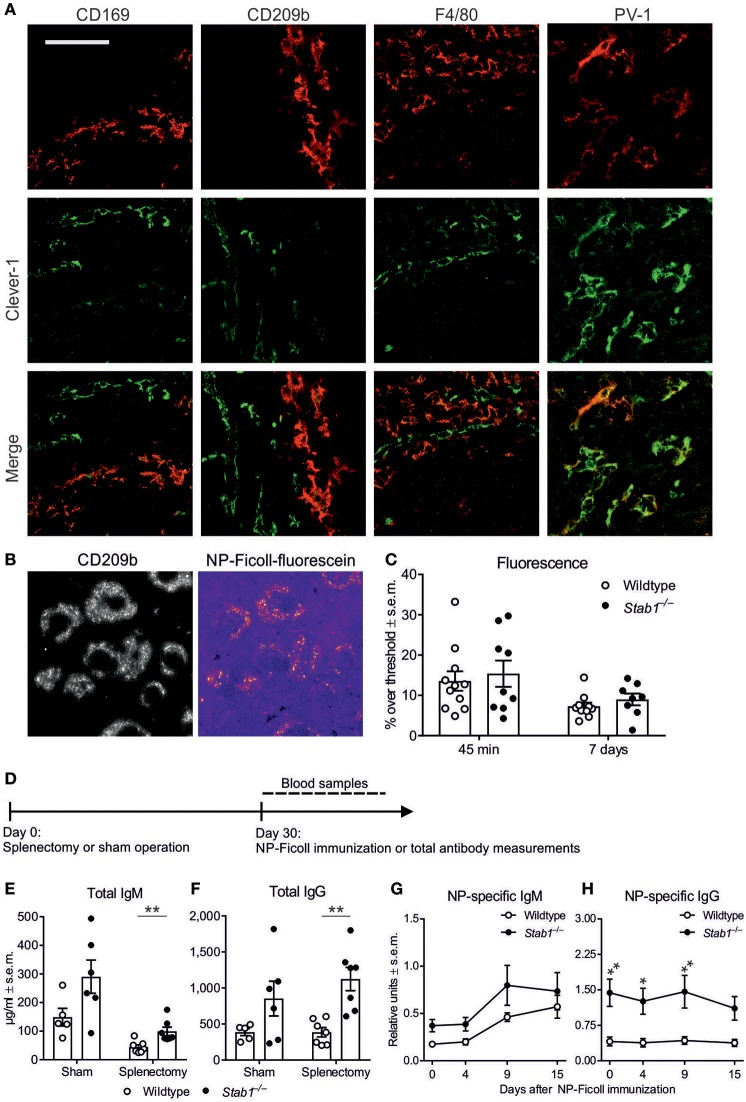
The elevated antibody levels in *Stab1*^−/−^ mice are regulated by extrasplenic factors. **(A)** Immunostaining of wildtype spleen sections with antibodies against Clever-1 (green, middle row) and CD169 (metallophilic macrophage marker), CD209b (MZ macrophage marker), F4/80 (red pulp macrophage marker) or PV-1 (vascular endothelial cell marker) (red, top row). Yellow color in the merged images indicates co-localization (bottom row) (scale bar, 200 μm). Images are representative of three independent experiments. **(B,C)** Representative immunostaining of fluorescein-labeled NP-Ficoll accumulation into CD209b^+^ MZ macrophages **(B)** and quantification at 45 min and 7 days **(C)** after intravenous injection. **(D)** Experimental protocol for splenectomy and NP-Ficoll immunization. **(E,F)** Plasma concentrations of IgM **(E)** and IgG **(F)** after splenectomy or sham operation as determined by ELISA. **(G,H)** Kinetics of NP-specific IgM **(G)** and IgG **(H)** production after NP-Ficoll immunization in splenectomized mice. **(C,E,F)** Each data point represents one mouse and bars represent the means ± s.e.m., *n* = 10–11 (wildtype) and 8–9 (*Stab1*^−/−^) **(C)** and 5–7 (wildtype) and 6–7 (*Stab1*^−/−^) **(E, F)**. **(G, H)** Data are presented as means ± s.e.m., *n* = 4 (wildtype and *Stab1*^−/−^). **(C,E–H)** Statistical significance was determined with Student's unpaired two-tailed *t*-test with Welch's correction when applicable **(C,E,F)** or two-way ANOVA followed by Bonferroni's multiple comparisons tests **(G,H)**, ^*^P < 0.05, ^**^P < 0.01.

Clever-1 is generally expressed by subsets of endothelial cells and immunosuppressive macrophages (2–5). In the spleen, however, we found that Clever-1 expression was strictly limited to PV-1^+^ vascular endothelial cells in the red pulp (Figure 3A). No anti-Clever-1 staining of spleen sections co-localized with CD209b^+^ marginal zone (MZ) macrophages, CD169^+^ metallophilic macrophages or F4/80^+^ red pulp macrophages (Figure 3A). The specificity of the anti-Clever-1 antibody was confirmed by the lack of reactivity with any cell type in spleens from *Stab1*^−/−^ mice (Supplementary Figure S2).

We then tested whether the accelerated antibody responses in *Stab1*^−/−^ mice might be attributed to better antigen access to the marginal zone due to systemically absent Clever-1–dependent scavenging. However, we saw no differences in the accumulation of intravenously injected NP-Ficoll into CD209b^+^ MZ macrophages between wildtype and *Stab1*^−/−^ mice after 45 min, reflecting immediate antigen access, or after 7 days, reflecting long-term antigen accumulation (Figures 3B,C). Thus, in the spleen, Clever-1 is exclusively expressed by vascular endothelial cells, and Clever-1 deficiency does not affect the delivery of intravascular TI antigen to the marginal zone.

To further analyze the possible contribution of the spleen to the altered humoral immune responses observed in *Stab1*^−/−^ mice, we studied antibody production in response to immunization after splenectomy (Figure 3D). As expected, splenectomy lowered basal IgM levels in both wildtype and *Stab1*^−/−^ mice when compared to sham-operated mice by 65.8 ± 14.9% and 70.1 ± 18.2%, respectively, whereas basal IgG levels remained unaffected (Figures 3E,F). Notably, even after splenectomy, the basal levels of IgM and IgG remained 123.5 ± 26.1% and 191.4 ± 39.0% higher, respectively, in *Stab1*^−/−^ mice compared to wildtype mice (*P* = 0.0059 and 0.0029, respectively) (Figures 3E,F). Additionally, after immunization with NP-Ficoll, splenectomized *Stab1*^−/−^ mice still produced NP-specific IgM more robustly than splenectomized wildtype mice, although these differences did not reach statistical significance (Figure 3G). Immunizing splenectomized mice did not further increase IgG levels in either genotype, but the IgG levels remained significantly higher in *Stab1*^−/−^ mice throughout the time course (Figure 3H). Our experiments on splenectomized mice therefore strongly suggest that the increased basal IgM and IgG and TI antigen–induced IgM levels in *Stab1*^−/−^ mice in comparison to wildtype mice are due to the effects of Clever-1 on B cell function mainly outside the spleen.

### Delayed B lymphopoiesis, B lymphocytemia and diminished peritoneal B1 cells in *Stab1*^−/−^ mice

In search of the extrasplenic origin of hyperimmunoglobulinemia in *Stab1*^−/−^ mice, we next analyzed B cell maturation in the bone marrow. Flow cytometric analysis revealed that the frequencies of pre-pro, pre and early mature B cells were reduced by 32.0 ± 3.4%, 35.9 ± 6.8% and 24.5 ± 0.7%, respectively, in *Stab1*^−/−^ mice compared to wildtype mice (*P* = 0.0140, 0.0002 and 0.0004, respectively), whereas those of late mature and transitional B cells were comparable between the two genotypes (Table 1). The frequencies of PC and T cell subpopulations in the bone marrow were comparable between wildtype and *Stab1*^−/−^ mice (Table 1 and Supplementary Table 1).

**Table 1:** Phenotype of B cells in wildtype and *Stab1*^−/−^ mice.

	**Wildtype**	***Stab1*^−/−^**	
**BONE MARROW**			
Pre-pro (B220^+^ CD24^low^ CD43^+^)	15.8 ± 1.1^a^ (*n* = 5)	10.7 ± 0.9^a^ (*n* = 5)	^*^^b^
Pre (B220^+^ CD24^+^ CD43^−^ IgM^−^ IgD^−^)	20.2 ± 2.1^a^ (*n* = 5)	13.0 ± 2.1^a^ (*n* = 5)	^***^^b^
Immature (B220^+^ CD24^+^ CD43^−^ IgM^low^ IgD^−^)	7.7 ± 0.2^a^ (*n* = 5)	7.7 ± 0.2^a^ (*n* = 5)	
Early mature (B220^+^ CD24^+^ CD43^−^ IgM^+^ IgD^+^)	27.9 ± 0.6^a^ (*n* = 5)	21.1 ± 0.4^a^ (*n* = 5)	^***^^b^
Late mature (B220^+^ CD24^+^ CD43^−^ IgM^low^ IgD^+^)	3.1 ± 0.2^a^ (*n* = 5)	4.4 ± 0.4^a^ (*n* = 5)	
Transitional (B220^+^ CD24^+^ CD43^−^ IgM^+^ IgD^−^)	9.9 ± 0.4^a^ (*n* = 5)	8.7 ± 0.6^a^ (*n* = 5)	
Plasma cells (CD45^+^ B220^low^ CD138^high^)	44.9 ± 2.5^a^ (*n* = 5)	44.1 ± 0.8^a^ (*n* = 5)	
B1 cells (CD19^+^ CD5^+/−^ CD11b^+^)	3.2 ± 0.5^a^ (*n* = 8)	3.4 ± 0.2^a^ (*n* = 9)	
**BLOOD**			
B220^+^ B cells	42.2 ± 5.3^a^ (*n* = 9)	60.6 ± 3.4^a^ (*n* = 9)	^*^^c^
**PERITONEAL CAVITY**			
B1 cells (CD19^+^ CD5^+/−^ CD11b^+^)	39.3 ± 4.1^a^ (*n* = 8)	22.4 ± 2.3^a^ (*n* = 9)	^**^^c^

a *% of cells (mean ± SEM)*.

b ^*^P < 0.05, ^***^P < 0.001 (two-way ANOVA).

c ^*^P < 0.05, ^**^P < 0.01 (Student's unpaired two-tailed t-test with Welch's correction).

In the blood, the frequency of B220^+^ B cells among lymphocytes was 43.5 ± 6.0% higher in *Stab1*^−/−^ mice compared to wildtype mice (*P* = 0.0147) (Table 1), whereas the frequency of CD4^+^ T cells was 38.8 ± 7.0% lower, (*P* = 0.0227) (Supplementary Table 1). Otherwise, the numbers of total leukocytes, lymphocytes and granulocytes in the blood of wildtype and *Stab1*^−/−^ mice (6.4 × 10^3^, 5.7 × 10^3^, and 0.67 × 10^3^ cells/μl in wildtype mice vs. 7.0 × 10^3^, 6.1 × 10^3^, and 0.75 × 10^3^ cells/μl in *Stab1*^−/−^ mice, respectively) were comparable between the two genotypes, as were the frequencies of total blood CD8^+^ T cells and blood CD4^+^ and CD8^+^ T cell subpopulations (Supplementary Table 1).

Because B1 cells also contribute to antibody production, we studied their abundance in the bone marrow and peritoneum in *Stab1*^−/−^ mice. This analysis revealed comparable frequencies of bone marrow B1 cells between wildtype and *Stab1*^−/−^ mice, but a 43.0 ± 6.2% decrease in the frequency of peritoneal B1 cells in *Stab1*^−/−^ mice compared to wildtype mice (*P* = 0.0032) (Table 1). Collectively, these data show alterations in early B cell lymphopoiesis in the bone marrow, in the numbers of circulating B cells and in peritoneal B1 cells in *Stab1*^−/−^ mice, yet these extrasplenic B cell aberrations do not apparently correlate with the hyperimmunoglobulinemia of these mice.

### Monocyte/macrophage Clever-1 regulates antibody production

**Figure 4:**
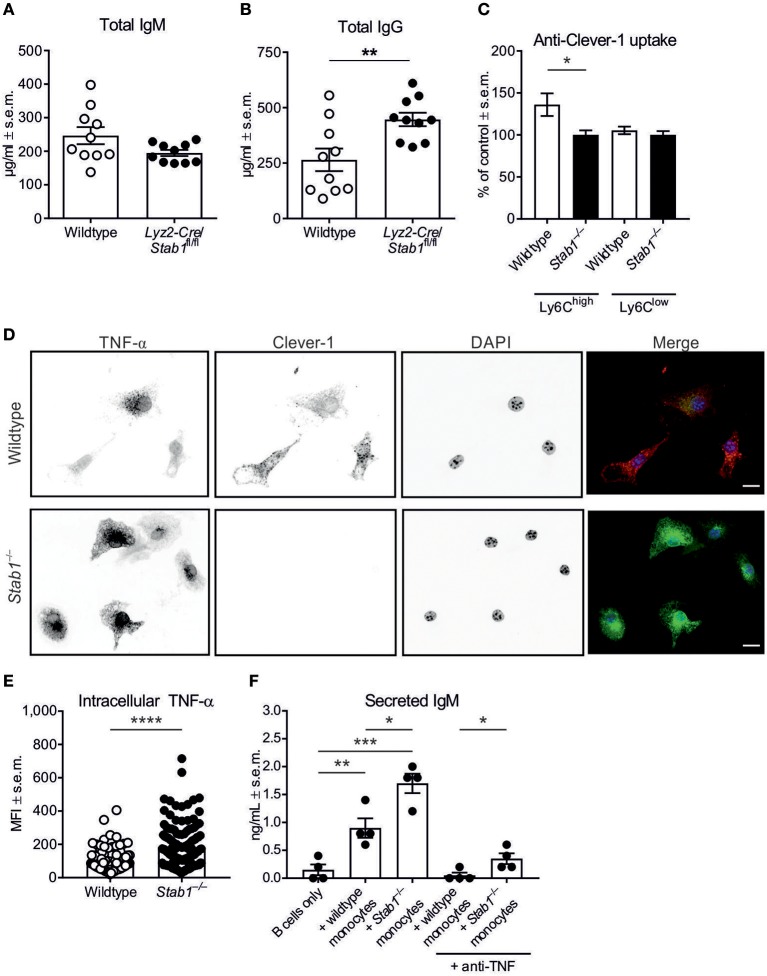
*Stab1*^−/−^ monocytes induce enhanced antibody production by B cells. **(A,B)** Baseline plasma concentrations of IgM **(A)** and IgG **(B)** in *Lyz2-Cre*/*Stab1*^fl/fl^ mice and control mice. **(C)** Uptake of fluorescently-conjugated anti-Clever-1 antibody by Ly6C^high^ and Ly6C^low^ monocytes from naïve mice. MFI values have been normalized to unspecific antibody uptake by the corresponding *Stab1*^−/−^ monocyte populations, which have been set at 100%. **(D)** Representative immunofluorescence images of wildtype and *Stab1*^−/−^ M2 macrophages treated with LPS and brefeldin A for 5 h. In the merged images, nuclei are shown in blue, TNF-α in green and Clever-1 in red (scale bar, 10 μm). **(E)** Quantification of TNF-α mean fluorescence intensities (MFI) per cell from **(D)**. The quantification is representative of three independent experiments. **(F)** Concentrations of secreted IgM as determined by ELISA from co-culture supernatants. Wildtype B cells were cultured with or without bone-marrow-derived monocytes/macrophages from wildtype or *Stab1*^−/−^ mice as indicated. **(A–C,E,F)** Each data point represents one mouse **(A–C)** or one cell **(E,F)** and bars represent the means ± s.e.m., *n* = 10 (wildtype and *Stab1*^−/−^) **(A, B)**, 4 (wildtype and *Stab1*^−/−^) **(C)**, 118 (wildtype) and 140 (*Stab1*^−/−^) **(E)** and 4 per condition **(F)**. Statistical significance was determined with Student's unpaired **(A–C,E)** or paired **(F)** two-tailed *t*-test with Welch's correction when applicable **(A)**, ^*^P < 0.05, ^**^P < 0.01, ^***^P < 0.001, ^****^P < 0.0001.

To analyze the potential role of macrophage Clever-1 in the regulation of antibody production, we utilized our *Lyz2-Cre*/*Stab1*^fl/fl^ mice that lack Clever-1 selectively from monocytes/macrophages (27). Notably, we observed increased plasma levels of IgG also in *Lyz2-Cre*/*Stab1*^fl/fl^ mice compared to wildtype mice (Figures 4A,B).

We have previously reported that in human monocytes/macrophages, silencing *Stab1* with siRNA and blocking Clever-1 with a specific antibody associate with the increased production of inflammatory cytokines, including TNF-α (15, 16). Incubation of peripheral blood leukocytes from wildtype and *Stab1*^−/−^ mice with directly-conjugated anti-Clever-1 antibody mStab1–1.26 or an isotype-matched control antibody revealed specific Clever-1 expression by Ly6C^high^ classical monocytes, but not by Ly6C^low^ non-classical monocytes (*P* = 0.0482) (Figure 4C). Moreover, bone-marrow-derived monocytes/macrophages from *Stab1*^−/−^ mice produced higher levels of TNF-α than those from wildtype mice when stimulated with LPS (*P* < 0.0001) (Figures 4D,E). Thus, murine monocytes and monocyte-derived macrophages express Clever-1, and Clever-1 is functionally involved in the regulation of TNF-α synthesis.

To determine whether the heightened inflammatory propensity of Clever-1-deficient monocytes/macrophages could be linked to the increased IgM production observed in *Stab1*^−/−^ mice, we performed co-culture experiments with wildtype splenic B cells (depleted of plasma cells) and wildtype or *Stab1*^−/−^ monocytes/macrophages isolated from bone marrow. Consistent with the fact that monocytes/macrophages regulate B cell function through the secretion of cytokines such as TNF-α (28, 29), ELISA of the co-culture supernatants showed increased secretion of IgM when either wildtype or *Stab1*^−/−^ monocytes were co-cultured with B cells compared to B cells alone (*P* = 0.0091 and 0.0002, respectively) (Figure 4F). Notably, *Stab1*^−/−^ monocytes were able to induce 47.1 ± 8.9% greater secretion of IgM by B cells compared to wildtype monocytes (*P* = 0.0171) (Figure 4F). The same effect was observed in another independent co-culture experiment (*P* = 0.0030). The increased IgM production was neutralized by TNF-α depletion in co-cultures with wildtype monocytes but only partially in co-cultures with *Stab1*^−/−^ monocytes, where the amount of secreted IgM remained significantly higher even after TNF-α depletion (*P* = 0.0321) (Figure 4F). Therefore, the contribution of other inflammatory factors whose production is increased in the absence of Clever-1 is likely. This notion is supported by the finding that interferon-activated gene 202b (*Ifi202b*) was upregulated in *Stab1*^−/−^ mice. RNA sequencing of liver samples from fetal and 2-weeks-old wildtype and *Stab1*^−/−^ mice showed that *Ifi202b* was not expressed in wildtype samples, but clearly expressed in samples from *Stab1*^−/−^ mice (log_2_ *Stab1*^−/−^/wildtype = 8.64 in fetal and 9.02 in 2-weeks-old mice) (Supplementary Table 2). Similarly, when assayed by qPCR from adult spleens, *Ifi202b* expression was negligible in wildtype mice but strongly induced in *Stab1*^−/−^ mice (RQ = 267, *n* = 3). Collectively, these experiments suggest that without Clever-1, monocytes/macrophages have an intrinsically heightened ability to stimulate B cells to produce antibodies.

## Discussion

We found here that *Stab1*^−/−^ mice manifest with elevated basal levels of IgM and IgG and augmented TD and TI antibody responses. While the frequency of B cells in the blood of *Stab1*^−/−^ mice was higher than in wildtype mice, the frequencies of B220^+^ B cells and FoB in the spleen were similar between the two genotypes, and the augmented antibody synthesis in the absence of Clever-1 was also observed in splenectomized mice. Therefore, Clever-1 does not seem to affect antibody production by controlling B cell numbers and humoral immune responses in the spleen. Instead, our functional data imply that Clever-1 in monocytes/macrophages controls the overall activity of B cells via the production of inflammatory cytokines.

*Stab1*^−/−^ mice are born at an expected Mendelian ratio and have no detectable macroscopic or microscopic defects or behavioral abnormalities (27, 30). Under physiological conditions, only a simultaneous deletion of Stab1 together with its close homolog Stab2 results in glomelurofibrotic nephropathy possibly by impairing the clearance of profibrotic cytokine GDF-15 in the liver (30). *Stab1*^−/−^ single-knockout mice show aggravated fibrosis and delayed resolution in liver injury models likely due to impaired myeloid-cell dependent removal of fibrogenic products of lipid peroxidation (25). These mice have also been shown to manifest with diminished tumor progression in melanoma, lymphoma or breast cancer models *in vivo*, probably due to altered migration of leukocytes to tumors and altered scavenging of certain extracellular matrix molecules (27, 31). *Stab1*^−/−^ mice do not manifest with opportunistic infections, and although they have not been subjected to infection models, short-term and long-term treatments of wildtype mice with function-blocking anti-Clever-1 antibodies did not result in increased rates of spontaneous infections or impairment in the clearance of experimental Staphylococcus aureus skin infections, arguing against an overall increase in the infection susceptibility in the absence of Clever-1 (12, 27).

Our current findings thus reveal for the first time the aberrant control of humoral immune responses in *Stab1*^−/−^ mice. Based on the well-known scavenging function of Clever-1, we first hypothesized that in the wildtype setting, antigen scavenging by Clever-1 might limit antigen delivery to B cells and consequently restrain humoral immune responses. Surprisingly, however, we found that in the spleen, Clever-1 was completely absent from all splenic macrophages, including red pulp, MZ and metallophilic macrophages, and was solely expressed by the vascular endothelium. The possible scavenging function of endothelial Clever-1 in the spleen and that of endothelial and macrophagic Clever-1 in other organs is highly unlikely, since the intravenously injected fluorescent TI-2 antigen NP-Ficoll accumulated similarly into MZ macrophages in both wildtype and *Stab1*^−/−^ mice. Moreover, splenectomy did not abrogate the constitutively higher IgM and IgG levels or the enhanced production of NP-Ficoll–specific IgM post-immunization in *Stab1*^−/−^ mice. Therefore, our data suggest that the exaggerated humoral immune responses in *Stab1*^−/−^ mice are largely of extrasplenic origins.

The enhanced humoral immune responses in *Stab1*^−/−^ mice are likely dependent on the altered functionality of monocytes/macrophages. This notion is supported by the finding that the increased baseline level of IgG was present also in mice in which Clever-1 is selectively deleted from these myeloid cell populations (conditional Stab1 knockout mice expressing Cre recombinase under the *Lyz2* promoter). We showed here also that the loss of Clever-1 in monocytes/macrophages enhances TNF-α production, and we have previously reported that in humans, Clever-1^high^ monocytes downregulate IFNγ and TNF-α production by antigen-specific T cells (16). Neutralizing TNF-α largely reduced the increased capacity of *Stab1*^−/−^ monocytes to support IgM secretion in co-culture experiments. Moreover, Stab1-/- mice manifested with a massive increase in *Ifi202b* transcription, an interferon-inducible gene (32). These data suggest that Clever-1 serves as an endogenous immunosuppressive molecule in monocytes/macrophages, where it maintains the production of inflammatory cytokines at physiological levels. When Clever-1 is removed from monocytes/macrophages, one consequence of the unrestrained production of inflammatory cytokines is the increased IgM and IgG production by B cells, possibly as a feedback regulatory response for compensating the actions of Clever-1.

Deleting or neutralizing Clever-1 augments antitumor immune responses at least in melanoma, lymphoma and breast cancer (27, 31). The efficacy of anti-Clever-1 antibodies in these experimental models has led to the development of fully human anti-Clever-1 antibodies that are entering clinical trials for cancer immunotherapy. Thus, our current observations suggest that disrupting the function of Clever-1 should not lead to the suppression of antibody production, and, in fact, could even improve humoral immune responses against tumor antigens.

## Ethics statement

All animal experiments were approved by the local Committee for Animal Experimentation in Finland (license numbers 5587/04.10.07/2014 and 5762/04.10.07/2017) and were performed according to the 3Rs principles and in accordance with the Finnish Act on Animal Experimentation (62/2006).

## Author contributions

JD, SJ, and MS conceived the study project and designed experiments. JD, MV, MK, PR, RV, KE, and MH performed experiments and analyzed the data. JD, MV, and MS drafted the manuscript. All authors contributed to the interpretation of the experiments, critically reviewed the manuscript, and gave final approval of the work.

### Conflict of interest statement

MH, SJ, and MS are shareholders in Faron Pharmaceuticals. The remaining authors declare that the research was conducted in the absence of any commercial or financial relationships that could be construed as a potential conflict of interest.
