# Supplementary material for: Enhanced Antibody Production in Clever-1/Stabilin-1–Deficient Mice
Source: Front Immunol. 2018 Oct 8;9:2257. doi: 10.3389/fimmu.2018.02257 (PMC6187969; doi:10.3389/fimmu.2018.02257)
Supplement: Supplementary file 1 [file Data_Sheet_1.pdf]

Enhanced Antibody Production in Clever-1/Stabilin-1–deficient mice

Johannes Dunkel[1†], Miro Viitala[1†], Marika Karikoski[1], Pia Rantakari[1], Reetta Virtakoivu[1], Kati Elima[1], Maija Hollmén[1], Sirpa Jalkanen[1] and Marko Salmi[1*]

Supplementary Figures

FIGURE S1. Splenic T cell phenotype in *Stab1*$^{-/-}$ mice. **(A–C)** Frequencies of splenic CD4^{+} and CD8^{+} T cells **(A)**, splenic CD4^{+} and CD8^{+} memory (CD44^{high} CD62L^{high}), effector (CD44^{high} CD62L^{low}) and naïve (CD44^{low} and CD62L^{high}) T cells **(B)** and splenic regulatory T cells (CD4^{+} CD25^{+} Foxp3^{+}) **(C)** in wildtype and *Stab1*$^{-/-}$ mice. **(A–C)** Each data point represents one mouse and bars represent the means ± s.e.m., n = 9 (wildtype and *Stab1*$^{-/-}$) **(A)**, 5 (*Stab1*$^{-/-}$ and wildtype) **(B)** and 7 (wildtype and *Stab1*$^{-/-}$) **(C)**. Statistical significance was determined with two-way ANOVA followed by Bonferroni's multiple comparisons tests **(A, B)** or Student's unpaired two-tailed *t*-test **(C)**, **** $p < 0.0001$.

FIGURE S2. The anti-Clever-1 antibody is specific. Sectioned spleens from wildtype (top row) and *Stab1*$^{-/-}$ mice (bottom row) were stained with antibodies against Clever-1 (left, green) or PV-1 (center, red). Merged images are shown on the rightmost column, yellow color indicates co-localization.

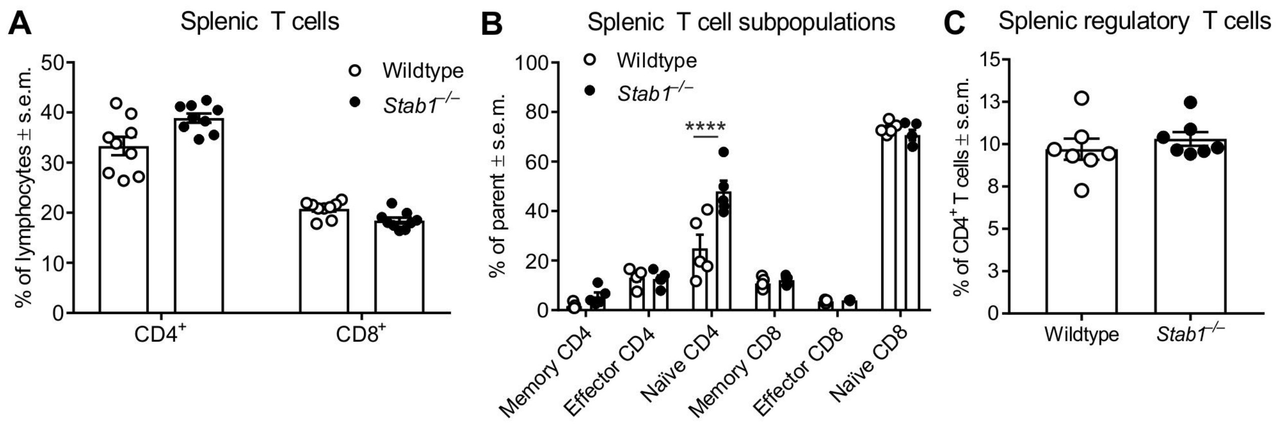

Supplementary Figure S1

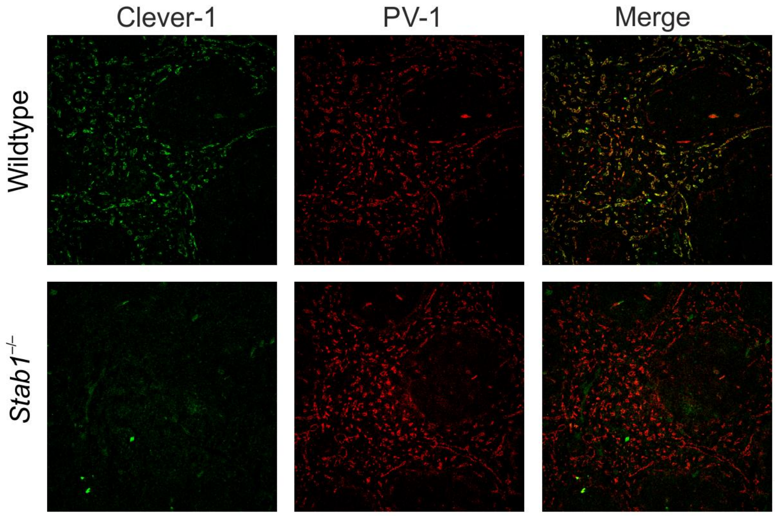

Supplementary Figure S2

Supplementary Tables

Supplementary Table 1. Phenotype of T cells in wildtype and *Stab1*$^{-/-}$ mice.

	Bone marrow		Blood		
	Wildtype	*Stab1*$^{-/-}$	Wildtype	*Stab1*$^{-/-}$	
CD4^{+} T cells					
Memory (CD44^{high} CD62L^{high})	21.8 ± 2.5[a)] (*n* = 5)	28.4 ± 1.3[a)] (*n* = 5)	6.9 ± 1.0[a)] (*n* = 4)	7.5 ± 1.7[a)] (*n* = 5)	
Effector (CD44^{high} CD62L^{low})	23.2 ± 2.0[a)] (*n* = 5)	22.0 ± 1.0[a)] (*n* = 5)	3.9 ± 1.1[a)] (*n* = 4)	3.2 ± 1.2[a)] (*n* = 5)	
Naïve (CD44^{low} CD62L^{high})	34.7 ± 5.0[a)] (*n* = 5)	27.3 ± 1.6[a)] (*n* = 5)	77.1 ± 3.9[a)] (*n* = 4)	70.0 ± 8.6[a)] (*n* = 5)	
Total			28.4 ± 3.6[a)] (*n* = 9)	17.4 ± 2.2[a)] (*n* = 9)	*[b)]
CD8^{+} T cells					
Memory (CD44^{high} CD62L^{high})	11.9 ± 1.6[a)] (*n* = 5)	13.4 ± 1.5[a)] (*n* = 5)	6.2 ± 1.5[a)] (*n* = 4)	5.2 ± 1.3[a)] (*n* = 5)	
Effector (CD44^{high} CD62L^{low})	6.9 ± 0.9[a)] (*n* = 5)	13.2 ± 2.5[a)] (*n* = 5)	6.5 ± 0.4[a)] (*n* = 4)	6.4 ± 0.6[a)] (*n* = 5)	
Naïve (CD44^{low} CD62L^{high})	55.1 ± 2.0[a)] (*n* = 5)	49.1 ± 2.1[a)] (*n* = 5)	33.0 ± 4.6[a)] (*n* = 4)	23.0 ± 7.8[a)] (*n* = 5)	
Total			29.4 ± 3.2[a)] (*n* = 9)	22.0 ± 1.5[a)] (*n* = 9)	

[a)] % of cells (mean ± SEM)
[b)]* $P < 0.05$, *** $P < 0.001$ (two-way ANOVA)

Supplementary Table 2.

List of significantly upregulated genes (FC > 2) in liver tissue from 2-week-old *Stab1*$^{-/-}$ mice compared to WT mice by RNA-seq analysis

Gene ID	$\log_2$ *Stab1*$^{-/-}$/WT	Symbol	Description
**26388**	**9,02**	**Ifi202b**	**interferon activated gene 202B**
624784	6,18	Gm9855	thymine DNA glycosylase pseudogene
19785	3,88	Rprl3	ribonuclease P RNA-like 3
619326	3,50	9130409I23Rik	RIKEN cDNA 9130409I23 gene
13097	3,42	Cyp2c38	cytochrome P450, family 2, subfamily c, polypeptide 38
13098	3,03	Cyp2c39	cytochrome P450, family 2, subfamily c, polypeptide 39
381806	3,03	Gm10319	murinoglobulin pseudogene
18405	2,92	Orm1	orosomucoid 1
629203	2,47	Sult2a3	sulfotransferase family 2A, dehydroepiandrosterone (DHEA)-preferring, member 3
546611	2,45	Klhl33	kelch-like 33
12053	2,30	Bcl6	B cell leukemia/lymphoma 6
17836	2,26	Mug1	murinoglobulin 1
12945	2,26	Dmbt1	deleted in malignant brain tumors 1
404195	2,11	Cyp2c54	cytochrome P450, family 2, subfamily c, polypeptide 54
667034	1,96	Pnp2	purine-nucleoside phosphorylase 2
545288	1,93	Cyp2c67	cytochrome P450, family 2, subfamily c, polypeptide 67
16625	1,86	Serpina3c	serine (or cysteine) peptidase inhibitor, clade A, member 3C
17835	1,85	Mug-ps1	murinoglobulin, pseudogene 1
20201	1,75	S100a8	S100 calcium binding protein A8 (calgranulin A)
103988	1,66	Gck	glucokinase
17837	1,65	Mug2	murinoglobulin 2
20202	1,64	S100a9	S100 calcium binding protein A9 (calgranulin B)
20714	1,63	Serpina3k	serine (or cysteine) peptidase inhibitor, clade A, member 3K

107141	1,56	Cyp2c50	cytochrome P450, family 2, subfamily c, polypeptide 50
17750	1,45	Mt2	metallothionein 2
1E+08	1,42	Gm15772	ribosomal protein L26 pseudogene
76933	1,38	Ifi27l2a	interferon, alpha-inducible protein 27 like 2A
12796	1,34	Camp	cathelicidin antimicrobial peptide
21786	1,32	Tff3	trefoil factor 3, intestinal
259301	1,28	Leap2	liver-expressed antimicrobial peptide 2
105171	1,28	Arrdc3	arrestin domain containing 3
331535	1,25	Serpina7	serine (or cysteine) peptidase inhibitor, clade A (alpha-1 antiproteinase, antitrypsin), member 7
14262	1,23	Fmo3	flavin containing monooxygenase 3
12012	1,23	Baat	bile acid-Coenzyme A: amino acid N-acyltransferase
26877	1,22	B3galt1	UDP-Gal:betaGlcNAc beta 1,3-galactosyltransferase, polypeptide 1
66425	1,20	Pcp4l1	Purkinje cell protein 4-like 1
54123	1,18	Irf7	interferon regulatory factor 7
66107	1,17	1100001G 20Rik	RIKEN cDNA 1100001G20 gene
629219	1,16	Sult2a6	sulfotransferase family 2A, dehydroepiandrosterone (DHEA)-preferring, member 6
21743	1,11	Inmt	indolethylamine N-methyltransferase
109054	1,10	Pfdn4	prefoldin 4
69142	1,10	Cd209f	CD209f antigen
76432	1,09	2310001H 17Rik	RIKEN cDNA 2310001H17 gene
13025	1,09	Ctla2b	cytotoxic T lymphocyte-associated protein 2 beta
14910	1,06	Gt(ROSA) 26Sor	gene trap ROSA 26, Philippe Soriano
53315	1,06	Sult1d1	sulfotransferase family 1D, member 1
68028	1,06	Rpl22l1	ribosomal protein L22 like 1
20115	1,05	Rps7	ribosomal protein S7
66121	1,04	Chchd1	coiled-coil-helix-coiled-coil-helix domain containing 1
78294	1,03	Rps27a	ribosomal protein S27A
50702	1,03	Cfhr1	complement factor H-related 1
69126	1,02	1810022K 09Rik	RIKEN cDNA 1810022K09 gene
108098	1,01	Med21	mediator complex subunit 21
20859	1,00	Sult2a1	sulfotransferase family 2A, dehydroepiandrosterone (DHEA)-preferring, member 1
59048	1,00	C1galt1c1	C1GALT1-specific chaperone 1

List of upregulated genes (FC > 2) in liver tissue from E17.5 *Stab1*$^{-/-}$ mice compared to WT mice by RNA-seq analysis			
**GeneID**	**log$_2$ *Stab1*$^{-/-}$/WT**	**Symbol**	**Description**
14915	11,04	Guca2a	guanylate cyclase activator 2a (guanylin)
99709	9,01	AI747448	expressed sequence AI747448
23844	8,85	Clca3	chloride channel calcium activated 3
20287	8,77	Sct	secretin

**26388**	**8,64**	**Ifi202b**	**interferon activated gene 202B**
1,01E+0 8	8,05	Mptx2	mucosal pentraxin 2
30060	7,90	Mfi2	antigen p97 (melanoma associated) identified by monoclonal antibodies 133.2 and 96.5
69826	7,57	Ms4a10	membrane-spanning 4-domains, subfamily A, member 10
66289	7,41	Mptx1	mucosal pentraxin 1
12591	7,22	Cdx2	caudal type homeobox 2
215384	7,12	Fcgbp	Fc fragment of IgG binding protein
68553	6,88	Col6a4	collagen, type VI, alpha 4
12590	6,84	Cdx1	caudal type homeobox 1
14120	6,72	Fbp2	fructose bisphosphatase 2
99662	6,57	Eps8l3	EPS8-like 3
14275	6,57	Folr1	folate receptor 1 (adult)
12557	6,25	Cdh17	cadherin 17
17287	6,18	Mep1a	meprin 1 alpha
19092	5,96	Prkg2	protein kinase, cGMP-dependent, type II
76768	5,95	Alpi	alkaline phosphatase, intestinal
22264	5,88	Prap1	proline-rich acidic protein 1
624681	5,86	Btnl6	butyrophilin-like 6
106407	5,84	Slc51a	solute carrier family 51, alpha subunit
545817	5,42	Cyp2w1	cytochrome P450, family 2, subfamily w, polypeptide 1
381352	5,28	Mamdc4	MAM domain containing 4
12716	5,25	Ckmt1	creatine kinase, mitochondrial 1, ubiquitous
65969	5,03	Cubn	cubilin (intrinsic factor-cobalamin receptor)
11808	4,97	Apoa4	apolipoprotein A-IV
20731	4,94	Spink4	serine peptidase inhibitor, Kazal type 4
23795	4,82	Agr2	anterior gradient 2 (Xenopus laevis)
546546	4,67	Serpina3h	serine (or cysteine) peptidase inhibitor, clade A, member 3H
239853	4,47	Gpr128	G protein-coupled receptor 128
93835	4,45	Amn	amnionless
226413	4,44	Lct	lactase
330440	4,33	Gm766	predicted gene 766
14916	4,31	Guca2b	guanylate cyclase activator 2b (retina)
72040	4,25	Cdhr5	cadherin-related family member 5
66260	4,24	Tmem54	transmembrane protein 54
11421	4,04	Ace	angiotensin I converting enzyme (peptidyl-dipeptidase A) 1
16763	3,97	Lad1	ladinin
54420	3,97	Cldn8	claudin 8
58860	3,67	Adamdec1	ADAM-like, decysin 1
22349	3,63	Vil1	villin 1
70008	3,59	Ace2	angiotensin I converting enzyme (peptidyl-dipeptidase A) 2
12797	3,57	Cnn1	calponin 1
11468	3,50	Actg2	actin, gamma 2, smooth muscle, enteric
17063	3,35	Muc13	mucin 13, epithelial transmembrane

69787	3,25	Anxa13	annexin A13
30962	3,23	Slc7a9	solute carrier family 7 (cationic amino acid transporter, y+ system), member 9
209195	3,20	Clic6	chloride intracellular channel 6
11768	3,13	Ap1m2	adaptor protein complex AP-1, mu 2 subunit
94071	3,09	Clec2h	C-type lectin domain family 2, member h
107753	3,06	Lgals2	lectin, galactose-binding, soluble 2
17075	3,04	Epcam	epithelial cell adhesion molecule
70163	3,03	Lypd8	LY6/PLAUR domain containing 8
14917	2,98	Gucy2c	guanylate cyclase 2c
381204	2,89	Naaladl1	N-acetylated alpha-linked acidic dipeptidase-like 1
16855	2,75	Lgals4	lectin, galactose binding, soluble 4
17880	2,60	Myh11	myosin, heavy polypeptide 11, smooth muscle
72273	2,55	2210404O 07Rik	RIKEN cDNA 2210404O07 gene
71960	2,55	Myh14	myosin, heavy polypeptide 14
20716	2,29	Serpina3n	serine (or cysteine) peptidase inhibitor, clade A, member 3N
14079	2,18	Fabp2	fatty acid binding protein 2, intestinal
239083	2,05	Ccnb1ip1	cyclin B1 interacting protein 1
74091	2,02	Npl	N-acetylneuraminate pyruvate lyase
75600	1,88	Calml4	calmodulin-like 4
53624	1,88	Cldn7	claudin 7
14778	1,79	Gpx3	glutathione peroxidase 3
84506	1,66	Hamp	hepcidin antimicrobial peptide
16669	1,59	Krt19	keratin 19
668489	1,50	Gm9199	glycine cleavage system protein H (aminomethyl carrier) pseudogene
212862	1,49	Chpt1	choline phosphotransferase 1
18242	1,49	Oat	ornithine aminotransferase
235504	1,48	Slc17a5	solute carrier family 17 (anion/sugar transporter), member 5
66438	1,41	Hamp2	hepcidin antimicrobial peptide 2
13809	1,39	Enpep	glutamyl aminopeptidase
19703	1,24	Renbp	renin binding protein
16006	1,23	Igfbp1	insulin-like growth factor binding protein 1
11475	1,19	Acta2	actin, alpha 2, smooth muscle, aorta
380780	1,14	Serpina11	serine (or cysteine) peptidase inhibitor, clade A (alpha-1 antiproteinase, antitrypsin), member 11
213393	1,10	8430408G 22Rik	RIKEN cDNA 8430408G22 gene
12091	1,05	Glb1	galactosidase, beta 1
